# The spectrum of resistance in SR/CR mice: the critical role of chemoattraction in the cancer/leukocyte interaction

**DOI:** 10.1186/1471-2407-10-179

**Published:** 2010-05-03

**Authors:** Gregory Riedlinger, Jonathan Adams, John R Stehle, Michael J Blanks, Anne M Sanders, Amy M Hicks, Mark C Willingham, Zheng Cui

**Affiliations:** 1 Department of Pathology, Wake Forest University School of Medicine, Medical Center Blvd, Winston-Salem, North Carolina, 27157, USA; 2 Department of Cancer Biology, Wake Forest University School of Medicine, Medical Center Blvd, Winston-Salem, North Carolina, 27157, USA; 3 Molecular Genetics and Genomics Program, Wake Forest University School of Medicine, Medical Center Blvd, Winston-Salem, North Carolina, 27157, USA

## Abstract

**Background:**

Spontaneous regression/complete resistance (SR/CR) mice are a unique colony of mice that possess an inheritable, natural cancer resistance mediated primarily by innate cellular immunity. This resistance is effective against sarcoma 180 (S180) at exceptionally high doses and these mice remain healthy.

**Methods:**

In this study, we challenged SR/CR mice with additional lethal transplantable mouse cancer cell lines to determine their resistance spectrum. The ability of these transplantable cancer cell lines to induce leukocyte infiltration was quantified and the percentage of different populations of responding immune cells was determined using flow cytometry.

**Results:**

In comparison to wild type (WT) mice, SR/CR mice showed significantly higher resistance to all cancer cell lines tested. However, SR/CR mice were more sensitive to MethA sarcoma (MethA), B16 melanoma (B16), LL/2 lung carcinoma (LL/2) and J774 lymphoma (J774) than to sarcoma 180 (S180) and EL-4 lymphoma (EL-4). Further mechanistic studies revealed that this lower resistance to MethA and LL/2 was due to the inability of these cancer cells to attract SR/CR leukocytes, leading to tumor cell escape from resistance mechanism. This escape mechanism was overcome by co-injection with S180, which could attract SR/CR leukocytes allowing the mice to resist higher doses of MethA and LL/2. S180-induced cell-free ascites fluid (CFAF) co-injection recapitulated the results obtained with live S180 cells, suggesting that this chemoattraction by cancer cells is mediated by diffusible molecules. We also tested for the first time whether SR/CR mice were able to resist additional cancer cell lines prior to S180 exposure. We found that SR/CR mice had an innate resistance against EL-4 and J774.

**Conclusions:**

Our results suggest that the cancer resistance in SR/CR mice is based on at least two separate processes: leukocyte migration/infiltration to the site of cancer cells and recognition of common surface properties on cancer cells. The infiltration of SR/CR leukocytes was based on both the innate ability of leukocytes to respond to chemotactic signals produced by cancer cells and on whether cancer cells produced these chemotactic signals. We found that some cancer cells could escape from SR/CR resistance because they did not induce infiltration of SR/CR leukocytes. However, if infiltration of leukocytes was induced by co-injection with chemotactic factors, these same cancer cells could be effectively recognized and killed by SR/CR leukocytes.

## Background

SR/CR mice are a unique colony of cancer-resistant mice derived from a single male BALB/c mouse that unexpectedly survived challenges with the extremely aggressive mouse cancer cell line S180 at doses up to several million times greater than the lethal dose for WT mice [1-3]. This resistance was remarkable since S180-induced malignancy with rapid lethality has not been effectively treated previously by any other existing therapy. The SR/CR trait was found to be inheritable in an autosomal dominant manner and has subsequently been bred into over 2000 descendants in several inbred mouse strains. This dominant trait of cancer resistance is mediated primarily by leukocytes of the innate immune system and does not require any prior manipulation. However, the trait could not be mapped to a specific chromosome region after numerous attempts by either backcross or congenic breeding strategies (unpublished results), suggesting that the responsible genetic element may not reside at a fixed chromosomal location. When SR/CR mice are challenged repeatedly with S180, the composition of infiltrating leukocytes remains primarily leukocytes of innate immunity [4]. The leukocytes of SR/CR mice can be transferred to sensitive wild-type (WT) mice for prevention and treatment of established malignancies in immune-compatible recipients without adverse side-effects [4].

Another intriguing property of the resistance in SR/CR mice is that they are healthy throughout their lifespan suggesting that normal cells in these mice are not harmed by the anticancer response that targets cancer cells with exceptionally high specificity [2]. The cancer resistant phenotype can be retained for life if the mice are frequently challenged with cancer cells. Isolated SR/CR leukocytes display *in vitro *cytolytic activity against a wide array of lethal transplantable mouse cancer cell lines that are distinct in origin, morphology and cellular properties [2,3]. Meanwhile, transformed but non-cancerous cells, such as CHO or NIH-3T3, are not killed by the SR/CR leukocytes *in vitro*[4].

Upon challenge with S180 tumor cells, the response of SR/CR mice to cancer cells involves three sequential yet distinct cellular processes of the leukocytes [5]. First, there is a rapid *infiltration *of SR/CR leukocytes to the site of cancer. This process requires the leukocytes to sense a chemoattractant gradient before making unidirectional movement from their storage sites, such as the bone marrow, spleen, peripheral lymph nodes and circulation, toward the higher end of the chemoattractant gradient. Meanwhile, a clearly-defined chemoattractant gradient must be established by cancer cells with the higher end of the gradient at the cancer site. Second, upon arrival at the cancer site, SR/CR leukocytes make tight *physical contact *with the surface of cancer cells, exemplified by rosette formation between cancer cells and leukocytes. This process requires SR/CR leukocytes to recognize unique surface properties of live cancer cells to allow surface binding between the plasma membranes of leukocytes and cancer cells. Third, upon surface contact, leukocytes deliver *effector mechanisms *to cause damage to the plasma membranes, swelling and eventual rupture of cancer cells. This final process involves, but is not limited to, several known common effector mechanisms, such as degranulation of neutrophilic granulocytes. The third process is not unique to SR/CR mice since these effector mechanisms have been reported previously in WT mice in the killing of cancer cells [6-8]. However, the first two processes were unique to SR/CR mice since WT leukocytes are unable to respond to the same cancer cells with either infiltration or rosette formation.

Based on the *in vitro *observations and other *in vivo *observations, this unique phenotype appears to be a true general resistance against cancer cells that may extend to additional mouse cancer cell lines. It would be helpful in elucidating the mechanism of SR/CR resistance if we were to identify cancer cell lines that could escape this resistance. Here, we report our findings.

## Methods

### Cell Lines and Mouse Strains

S180, EL-4, LL/2, B16, and J774 cell lines were purchased from American Type Culture Collection and propagated in culture according to the manufacturer's protocol. MethA were a kind gift from Dr. Lloyd Old (Ludwig Institute for Cancer Research, New York). BALB/c mice were purchased from Charles River Laboratory and C57BL/6 mice were purchased from the Jackson Laboratory. SR/CR mice (1, 4) were bred at the Animal Research Programs of Wake Forest University (WFU) Health Sciences. Mice were housed in plastic cages covered with individual air filter tops, containing corncob shavings as bedding, allowed free access to water and regular chow and exposed to a 12-hr fluorescent light/dark cycle. All animal procedures were conducted according to Institutional Animal Care and Use Committee guidelines and the National Institutes of Health Guide for the Care and Use of Animals and with all protocols and procedures approved by the IACUC of the WFU Health Sciences. Mature mice (~2-6 months old) were used for all experiments unless specifically noted elsewhere.

### SR/CR *In Vivo *Resistance

SR/CR and WT mice were injected with the indicated number of cancer cells i.p. and the ability of mice to resist the cells was determined by survival. Moribund mice were euthanized. Cell-free ascites fluid (CFAF) was obtained by collection of ascites fluid from WT mice that developed S180 induced ascites. This ascites fluid was spun twice at 400 g for 5 minutes each, followed by one spin at 3000 g for 5 minutes with the cell free supernatant collected after each spin. This CFAF was then used fresh for subsequent experiments. In the co-injection experiments, 2 ml of CFAF was co-injected with MethA or LL/2 and these mice then received weekly injections of CFAF for the duration of the experiment.

### Immune Infiltration in Response to Cancer Cell Lines and CFAF

SR/CR or WT mice were injected i.p. with the indicated cancer cell line or CFAF. Heat inactivated CFAF was obtained by heating CFAF for 15 minutes at 100°C in a water bath. Fractions of CFAF containing molecules > or < 5 kD were obtained using Amicon Ultra 5 k centrifugal devices (Millipore, Billerica, MA) according to the manufacturer's instructions. Briefly, total CFAF was placed in the upper chamber of the centrifugal device and then spun at 3000 g for 20 minutes separating the > 5 kD fraction into the top chamber and the < 5 kD fraction into the bottom of the tube. A heat inactivated sample of the < 5 kD fraction was obtained by heating this fraction for 15 minutes at 100°C in a water bath. Six hours after injection the peritoneal cavity was lavaged and the number of cells was quantitated using a cytometer. For each injection group, n >= 4.

### Flow Cytometry

Cells from peritoneal lavages were stained with specific antibodies to the cell surface markers Ly6G, NK1.1, F4/80, CD11c, CD19, CD4, and CD8 (BD Pharmingen, San Diego, CA) according to standard procedures recommended by Pharmingen. Briefly, 400,000 cells from the peritoneal lavage were added in 100 μl of FMF medium (PBS w/1% FBS) in 12 × 75 mm flow tubes that were spun at 400 g × 5 minutes. The supernatant was removed and the cells were resuspended in 50 μl of 5 μg/ml FITC-conjugated antibody (Ly6G, NK1.1, F4/80, CD11c, CD19, CD4, or CD8) or FMF medium alone as a control and incubated 30 minutes on ice in the dark. The cells were then washed twice with 100 μl of FMF medium and then resuspended in 300 μl of FMF medium keeping the samples in the dark as much as possible. The samples were then analyzed on a FACSCalibur flow cytometer (BD Bioscience, Mountain View, CA). Forward and side scatter gain settings were tuned to sort live cells from cell fragments. The total value for these seven markers was arbitrarily set at 100% for each sample to allow comparison between groups and for each injection group n >= 4. S180, MethA, and LL/2 were each tested and found to have no reactivity to any of the cell surface markers used.

## Results

### SR/CR Mice Resist a Broad Array of Lethal Mouse Cancer Cell Lines

**Figure 1:**
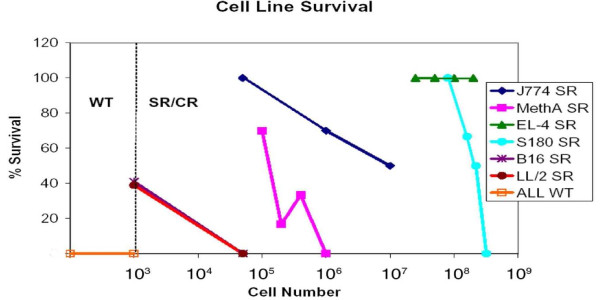
**SR/CR mice resist a broad range of lethal cancer cell lines at different doses**. The percentage of SR/CR mice that were able to survive challenge with J774, MethA, EL-4, S180, B16, or LL/2 was determined empirically. Mice were monitored for at least 60 days after challenge. SR/CR C57BL/6 mice were challenged with 10e3 or 5 × 10e4 B16 or LL/2. SR/CR C57BL/6 mice were also challenged with 0.8 × 10e7, 1.6 × 10e8, 2.2 × 10e8, or 3.2 × 10e8 S180 or 2.5 × 10e7, 5 × 10e7, 10e8, or 2 × 10e8 EL-4. SR/CR BALB/c mice were challenged with 10e5, 2 × 10e5, 4 × 10e5, or 10e6 MethA or 5 × 10e4, 10e6, or 10e7 J774. WT mice uniformly died at all doses with all cell lines tested. At least 5 mice were tested for any given dose of tumor cells injected.

To determine the respective maximum tolerated doses (MTD), we challenged SR/CR mice and control WT mice with various lethal mouse cancer cell lines at different doses and measured the disease-free survival of the mice. SR/CR mice were first identified by their ability to survive 2 standard screens with 2 × 10e5 and 5 × 10e6 S180 i.p., which always induced lethality in WT mice. The mice were then divided into groups that were challenged with other cancer cells at different doses. SR/CR mice in the BALB/c background were challenged with J774 lymphoma (MHC haplotype H2d) or MethA sarcoma (H2d) cells. SR/CR mice in the C57BL/6 congenic background (generations n8 or later) were challenged with EL-4 lymphoma (H2b), LL/2 lung carcinoma (H2b), B16 melanoma (H2b) or S180 sarcoma (H2q). All WT mice died with i.p. doses of 10e3 cells or fewer with all cancer cell lines tested. The MTD of LL/2 for WT mice was less than 100 cells. As few as one viable EL-4 has been reported previously to be lethal in WT mice [9]. In the BALB/c background, all SR/CR mice survived 5 × 10e4 J774 before they began to die at higher doses, while 70% of mice challenged with 10e5 MethA survived. In the C57BL/6 background, all SR/CR mice survived with 8 × 10e7 S180 before they began to die at higher doses. No death in SR/CR mice was observed with doses up to 2 × 10e8 for EL-4. The survival of C57BL/6 SR/CR mice challenged with B16 and LL/2 was greatly reduced with 40% surviving when challenged with 10e3 cells in both groups (Figure 1). Although no mice survived challenge with B16 or LL/2 at higher doses, the average survival time of SR/CR mice at 5 × 10e4 B16 was greatly increased (~34 days) compared to WT mice (~21 days).

### Resistance of naïve SR/CR pups to cancer cells other than S180

**Figure 2:**
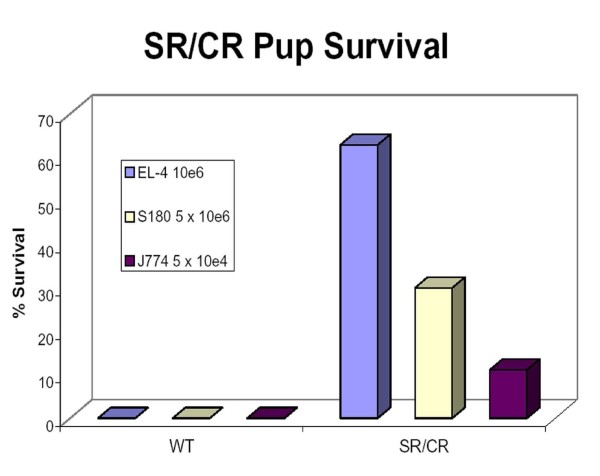
**Naïve SR/CR pups resist the initial challenge with EL-4 and J774**. Pups from an SR/CR × WT cross were challenged with 10e6 EL-4 prior to being challenged with any other cancer cell line and 22 out of 35 pups survived. Pups from an SR/CR × WT cross that were challenged with 5 × 10e5 and a second challenge with 5 × 10e6 S180 have a historical survival rate of ~30% [1,2]. When nine pups from an SR/CR × WT cross were challenged with 5 × 10e4 J774 prior to being challenged with any other cell line, one mouse survived. All WT mice challenged with EL-4, S180, and J774 at the indicated doses uniformly died. Mice were monitored for at least 60 days after challenge.

We injected naïve pups, age 6-8 weeks, with cancer cell lines other than S180 initially to determine if resistance to these cell lines was simply a result of cross-vaccination from antigens shared with S180 or if it was a result of innate recognition of a common surface property shared between different cancer cell lines. SR/CR mice had never previously been tested for their ability to resist additional cancer cell lines without first being able to survive challenge with S180. The naïve pups were obtained from our routine breeding scheme in which one C57BL/6 SR/CR parent is crossed with a C57BL/6 WT parent. In this breeding scheme, 30-40% of the naive pups were expected to survive the initial challenge with S180. Five weeks after the naïve pups were given 10e6 EL-4 as their first cancer challenge, 22 of 35 pups (65%) survived and remained apparently healthy (Figure 2). When naïve pups were first screened with J774 at 5 × 10e4, 1 of 9 pups survived.

### S180 or CFAF Enhances the Resistance of SR/CR mice to MethA and LL/2

**Figure 3:**
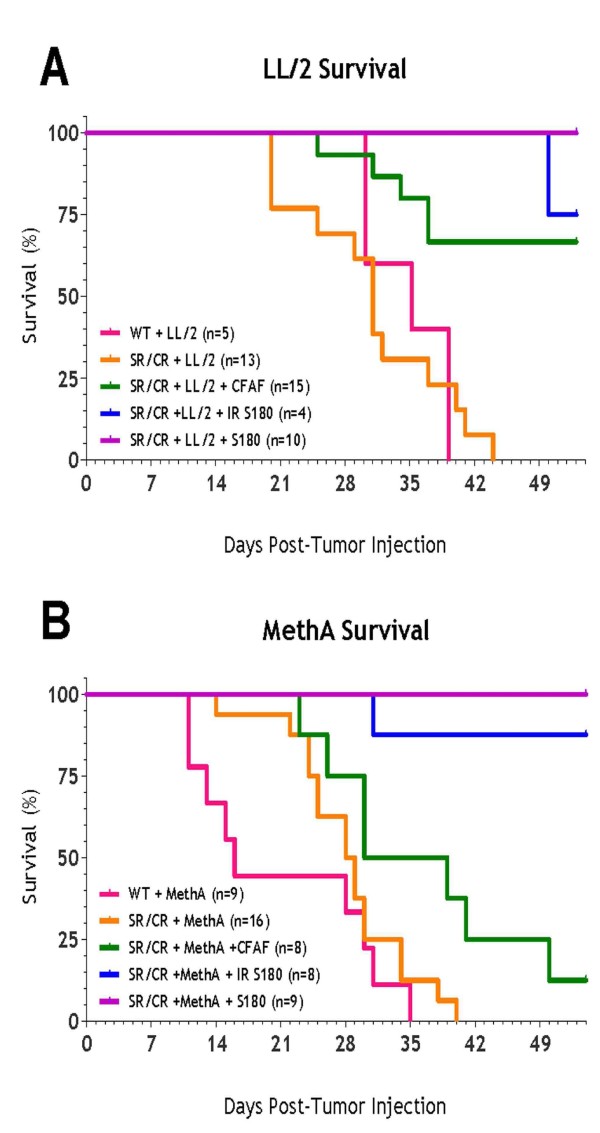
**S180 or cell free ascites fluid (CFAF) enhances SR/CR resistance**. SR/CR or WT mice were challenged with 2 × 10e5 LL/2 (upper panel) or 10e6 MethA (lower panel) i.p. either alone or with 10e6 S180, 10e6 irradiated S180, or weekly injections of CFAF and survival curves were generated. The number of mice in each group is indicated in the figure.

It was somewhat surprising that the resistance of SR/CR mice against some other cancer cell lines was so much weaker than that against S180 and EL-4, whereas our in vitro assay results show that the SR/CR leukocytes could kill all of these cancer cell lines with similar efficiencies [[2], Z.C. unpublished results]. The increased sensitivity of SR/CR mice to MethA and LL/2 suggested that a failure might have occurred at one or more of the three required stages of leukocyte action for the anticancer response. We set out to examine which of the three required stages of leukocyte action was responsible for this failure of the anticancer response. If the increased sensitivity was caused by a failure of LL/2 cells in immune attraction, the resistance should be restored in SR/CR mice when LL/2 were co-injected with live cancer cells that were known to induce leukocyte infiltration, such as S180. To test this hypothesis, we mixed 2 × 10e5 LL/2 cells with 10e6 S180 cells and co-injected them i.p. into SR/CR mice. All of the co-injected SR/CR mice survived, whereas all of the co-injected WT mice died. A nearly identical result was achieved when LL/2 was co-injected with 10e6 irradiated S180 (25 Gy) that were alive for several days but were not proliferating. To further test the hypothesis that the resistance to LL/2 at 2 × 10e5 was restored by simply provoking leukocyte migration, we examined if S180-induced CFAF could achieve the same effect as the live or irradiated S180. CFAF (cell-free ascites fluid) is the immediate liquid environment produced as the S180 cells grow in the peritoneum of WT mice. If the enhanced survival from the co-injection was truly mediated by leukocyte migration as a result of diffusible chemoattractants from S180, CFAF should contain all the diffusible chemoattractants and should be able to recapitulate the enhanced survival observed with live S180 cells to some extent. Indeed, when 2 × 10e5 LL/2 were co-injected with CFAF (with 2 ml weekly), a clear survival benefit was observed (Figure 3** upper panel**). A similar result was obtained for the resistance to MethA co-injected with S180, irradiated S180 and CFAF (Figure 3** lower panel**) and preliminary results indicate that S180 also enhances the survival of SR/CR mice challenged with higher doses of B16 (data not shown).

### S180 only enhances the resistance of SR/CR mice to LL/2 locally

**Figure 4:**
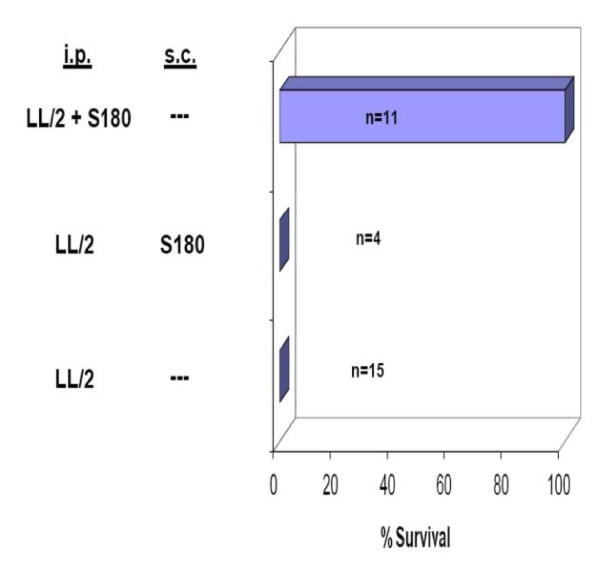
**S180 only enhances SR/CR resistance locally**. SR/CR mice were challenged with 2 × 10e5 LL/2 *i.p*. either alone or with 10e6 S180, either *i.p*. or *s.c*., and the survival of mice was monitored. The number of mice in each group is indicated in the figure.

The improved survival in SR/CR mice co-injected with S180 or CFAF could be the consequence of two distinct events; higher infiltration of SR/CR leukocytes or improved effector function by systemic activation of SR/CR leukocytes induced by S180 coinjection. To determine which of the processes predominated, we examined the ability of S180 to enhance SR/CR resistance to LL/2 when injected in the same site or at a remote site (Figure 4). Our results indicate that S180 only enhances resistance to LL/2 when injected at the same site.

### S180 and CFAF induce leukocyte infiltration in SR/CR mice

**Figure 5:**
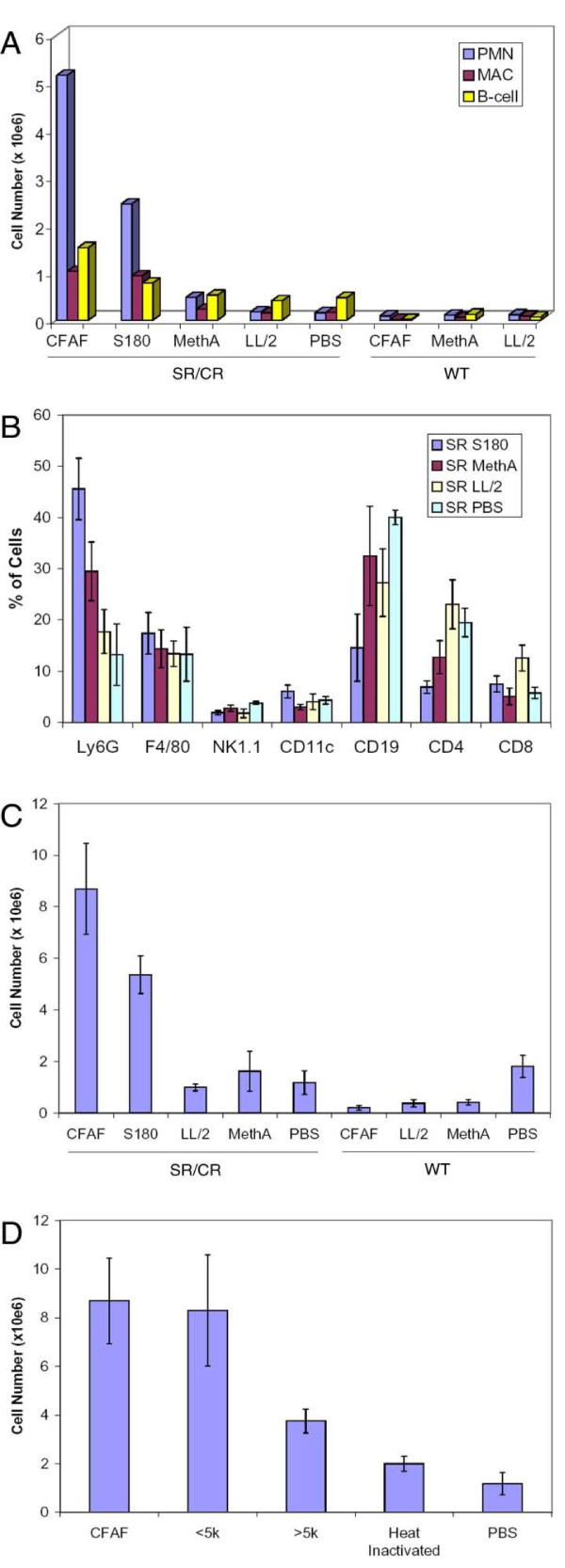
**Leukocyte subset composition during challenge with different cancer cell lines**. A) SR/CR and WT mice were challenged with 10e5 cells of the indicated cancer cell line, CFAF, or PBS and responding leukocytes were washed out at six hours, quantitated, stained with Ly6 g (PMN), F4/80 (Mac), and CD19 (B-cell) and profiled by flow cytometry. B) Composition of infiltrating immune cells. SR/CR mice were injected with 10e5 cells of the indicated cancer cell line or PBS and six hours later the peritoneal cavity was washed out. The peritoneal infiltrate was stained with Ly6 g (PMN), F4/80 (Mac), NK1.1 (NK cell), CD11c (dendritic cell), CD19 (B-cell), CD4, and CD8 and profiled by flow cytometry. The total value for these seven markers was arbitrarily set at 100% for each sample to allow comparison between groups. Values represent the mean +/- SEM. For each injection group, n >= 4. C) Immune infiltration in response to cancer cell lines and CFAF. SR/CR or WT mice were injected i.p. with 10e5 cells of the indicated cancer cell line or CFAF and six hours later the peritoneal cavity was washed out and the number of cells was quantitated using a cytometer. Values represent the mean +/- SEM. For each injection group, n >= 4. D) SR/CR immune infiltration in response to CFAF, heat inactivated CFAF, and small (<5 kD) or large (>5 kD) molecules found in CFAF fluid. SR/CR mice were injected as indicated and six hours later the peritoneal cavity was washed out and the number of cells was quantitated using a cytometer. Values represent the mean +/- SEM. For each injection group, n >= 4.

The need for co-injection suggested that the ability to induce leukocyte infiltration along with the number of infiltrating leukocytes may be directly related to the number of cancer cells the SR/CR mice can resist, with a strong infiltration resulting in a higher MTD. We hypothesized that the low-MTD cancer cells (LL/2 and MethA) should have lower activity for inducing SR/CR leukocyte infiltration, whereas the high-MTD cancer cells (S180) should have higher activity for inducing leukocyte infiltration. To test this hypothesis, we quantified the number of infiltrating cells in the peritoneum and used flow cytometry to profile the cell types of the infiltrating leukocytes based on surface markers six hours after being challenged with different cancer cells. Aliquots of leukocytes were specifically labeled with surface antibodies for neutrophils (Ly6G positive), macrophages (F4/80 positive), natural killer cells (NK1.1 positive), dendritic cells (CD11c positive), B cells (CD19 positive), helper T cells (CD4 positive), and cytolytic T cells (CD8 positive) cells and then subjected to analysis by flow cytometry. The sum of these seven markers was arbitrarily set at 100% to allow comparison between different groups. The most noticeable differences were in the numbers of neutrophils and macrophages in response to S180 or CFAF between WT and SR/CR mice, while levels of B cells remained fairly consistent (Figure 5A). It was clear that S180 induced significantly more leukocyte infiltration in SR/CR mice than MethA and LL/2, and the percentage of leukocytes was shifted towards innate immune cells, specifically neutrophils and macrophages. It was interesting to note that this effect was specific for SR/CR mice since cancer cells or CFAF appeared to have an inhibitory effect on WT leukocyte infiltration (Figure 5C). CFAF provoked the largest leukocyte infiltration, consistent with the idea that the highest concentration of diffusible chemoattractants would accumulate in the peritoneal fluid over several days in the presence of a large number of live S180 cells. We also characterized some of the biochemical properties of the chemoattractants in S180-induced CFAF. When CFAF was fractioned through an Amicon Ultra 5 k centrifugal device, most of the chemoattraction activity was recovered in the filtered-through fraction, suggesting that the chemoattractants were smaller than 5 kilo-Dalton. Furthermore, boiling CFAF for 10 min abolished most of the chemoattracting activity, suggesting that the chemoattractants were heat sensitive (Figure 5D).

## Discussion

In the present study, we characterized the ability of SR/CR mice to resist additional lethal cancer cell lines *in vivo*, and showed that SR/CR mice are able to resist these cell lines at higher doses than WT mice. However, SR/CR mice were able to resist some cancer cell lines at exceptionally high doses (high-MTD), whereas other cancer cell lines were only resisted at moderate or lower doses (low-MTD). We found that co-injection with either S180 or CFAF, both capable of inducing massive leukocyte infiltration specifically in SR/CR mice, was able to increase the level of resistance of SR/CR mice to cell lines with otherwise low-MTD.

Cancer cells may undergo selection to avoid detection by the immune system, a process termed cancer immuno-editing that may drive the progression of malignancy [10]. A variety of tumor-derived factors may contribute to immunosuppressive processes that may extend immune evasion from the primary site to peripheral sites in patients with cancer [11]. The results from our experiments suggest that cancers with low-MTD escape from SR/CR anticancer immunity because they do not produce sufficient chemoattractants. These low-MTD cancer cell lines are capable of being eradicated in SR/CR mice at lower doses, presumably by the less than 2 × 10e6 resident leukocytes usually present locally in the peritoneal cavity. At higher doses, however, the growth of these low-MTD cancer cell lines may outpace the killing ability of these limited resident leukocytes. This is in contrast to cell lines such as S180 that are able to induce a very large infiltration of leukocytes. The larger numbers of infiltrating SR/CR leukocytes are apparently capable of killing a much larger number of cancer cells.

**Figure 6:**
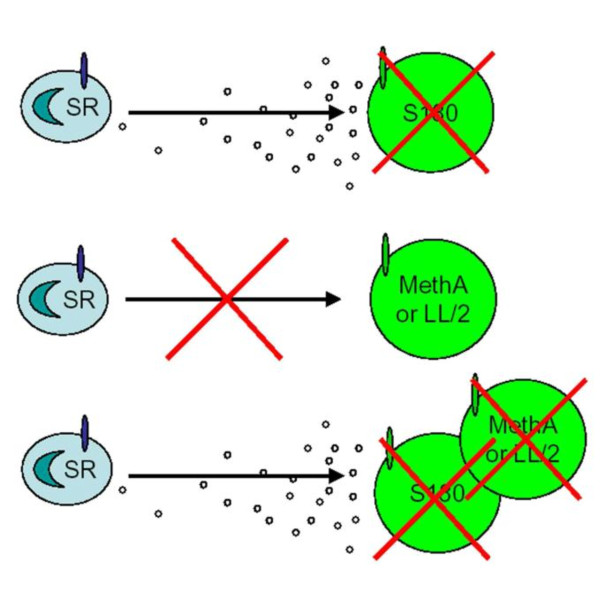
**SR/CR leukocyte migration and cancer destruction**. A proposed model for SR/CR mediated cancer cell killing. S180 secretes chemotactic factors that attract SR/CR leukocytes (SR) to the site of the cancer, allowing tight contact between the leukocytes and cancer cells to be made which facilitates tumor destruction. LL/2 and MethA produce little or no chemoattractant and are, therefore, not efficiently killed by SR/CR leukocytes. Co-injection of MethA or LL/2 with S180 results in attraction of SR/CR leukocytes to the site of the cancer and efficient cell killing of these cancer cell lines. This suggests that some cancer cells may escape from SR/CR resistance because they do not induce SR/CR leukocyte infiltration and demonstrates the importance of leukocyte infiltration in the SR/CR resistance mechanism.

Although the outcome of the co-injection experiment with S180 and CFAF was somewhat unexpected, the results, coupled with our previous work demonstrating that S180, MethA, and LL/2 were killed in an *in vitro *assay [2], suggest that the ability to induce leukocyte infiltration may be the most significant factor between these cancer cells in their ability to be resisted by SR/CR mice. There appear to be common surface properties that allow a variety of cancer cells to be recognized, bound, and destroyed by SR/CR leukocytes when they are in close proximity (Figure 6). Our results demonstrate that S180 only enhances resistance against LL/2 locally in SR/CR mice and argues that the ability of S180 to induce leukocyte infiltration is the critical event in augmenting resistance. While immune infiltration is clearly important for the eradication of cell lines such as LL/2 and MethA, we cannot completely exclude the possibility that co-injection with S180 or CFAF also activates the effector mechanism of SR/CR leukocytes, in addition to the induction of their infiltration.

The results also suggest that there is a diffusible chemoattractant gradient established by some cancer cells. CFAF from S180 contains high levels of these chemoattractants that are specific for SR/CR leukocytes. Our results indicate that the most active chemoattractants are molecules smaller than 5 kD that are heat sensitive. It is currently unclear if these diffusible chemoattractants were produced by active cellular secretion [11,12], by passive "surface shedding", a physical process, from cancer cells [13-16] or indirectly by the interaction between cancer cells and stromal tissues. Nevertheless, CFAF offers a good platform for further biochemical purification and identification of these chemoattractants. Apparently, chemoattraction of SR/CR leukocytes is a separate process from recognition of the common cancer cell surface properties that allow for local binding and eradication of SR/CR leukocytes, since some cancer cells can lose the former process while retaining the properties of the latter. This ability to induce leukocyte infiltration through chemoattraction appears to be the reason that SR/CR leukocytes are effective as a systemic therapy against established S180 cancers, but are only locally effective against cancers like LL/2.

The cancer/immune cell interaction involves events on either side that influence the ability of the immune system to eradicate the cancer. On the cancer side, malignant lesions may range from being highly immune-attractive (S180) to inducing little immune infiltration (LL/2). Cancer cells may develop mechanisms that prevent migration of leukocytes to the site of the cancer, either by turning off the production of inflammatory molecules that can act as chemoattractants or by producing molecules that actively inhibit immune cells, such as TGF-β [17]. Our results indicate that cancer cells and CFAF may also produce molecules that are directly inhibitory to WT leukocytes (Figure 4C). On the host side, leukocytes themselves can vary significantly in any of the three required stages of leukocyte response. If leukocytes have a defect in infiltration to the cancer site, recognition of the cancer, or deployment of their effector mechanisms an effective anticancer response to protect the host cannot take place. For example, leukocytes may infiltrate the cancer site but may be unable to recognize and kill the cancer cells. This has been reported in some melanoma patients, in whom despite having melanoma-specific T cells infiltrating the tumor lesions, tumor rejection rarely occurs [18]. Additionally, the responsiveness of host leukocytes may be influenced by genetics, aging and environmental factors.

Our SR/CR model system is very interesting in light of many recent reports that there is a positive correlation between tumor infiltrating lymphocytes and the survival of patients with melanoma, ovarian cancer, bladder cancer, glioma, and colon cancer [19-23]. It is worth noting that in these examples of human cancers the correlation is with cells of the adaptive immune system, specifically T lymphocytes, while in the SR/CR mouse the resistance mechanism is mediated by the innate immune system. A recent study performed by Galon *et al*. is particularly intriguing as it specifically links activation of the cellular immune response, including macrophages of the innate immune system, to patient outcomes in colorectal cancer [24]. They find a significant correlation between expression of genes of the Th1 response and a beneficial outcome in risk of relapse after complete removal of the tumor.

The best case scenario for an anticancer protection mechanism in a host would be having cancer cells that secrete a chemoattractant and having leukocytes that can infiltrate, recognize the cancer cells as foreign, and completely destroy the cancer. However, the absence of one or more of these factors could lead to an unfavorable host/cancer interaction enabling the cancer to escape immunosurveillance resulting in progression of the disease. When there is no recognition of cancer cells by leukocytes, manipulation of other processes, such as infiltration, will not improve host survival. However, if infiltration is the only deficiency, as we observe in SR/CR mice challenged with LL/2, local delivery of host leukocytes or establishment of a chemoattractant gradient at the cancer site could achieve therapeutic benefit.

## Conclusions

Our results show that SR/CR mice had significantly higher resistance to all cancer cell lines tested in comparison to wild type (WT) mice. However, there was great variation in the number of cells that could be resisted by SR/CR mice across the different cancer cell lines tested. It appears that this variation is based on the ability of the cancer cell lines to induce leukocyte infiltration, as co-injection with chemoattractant factors increased the number of cancer cells that the SR/CR mice could resist. We also demonstrate for the first time that SR/CR mice are able to resist cell lines other than S180 on the initial challenge. Therefore, it appears that the resistance against other cancer cells is also innate in nature since it does not require specific priming with non-self antigens found on S180. This supports the idea that SR/CR mice innately recognize a factor that is common on multiple cancer cell lines but absent on non-neoplastic cells.

## Competing interests

The authors declare that they have no competing interests.

## Authors' contributions

GR helped conceive of the study, participated in its design and coordination, helped perform the injections, lavages, flow cytometry, and drafted the manuscript. JA helped perform the injections, lavages, animal care and helped with manuscript preparation. JS helped design the studies, perform the lavages, irradiation, flow cytometry, and with manuscript and figure preparation. MB helped design the studies, perform the lavages/injections, and with manuscript and figure preparation. AS helped design the studies, perform the lavages/injections, and with manuscript preparation. AH helped design the studies, perform the lavages/injections, and with manuscript preparation. MW helped design the studies, helped with the histological evaluation, and with manuscript preparation. ZC was responsible for the oversight of the entire project and including experimental design and manuscript writing. All authors read and approved the final manuscript.

## Pre-publication history

The pre-publication history for this paper can be accessed here:

http://www.biomedcentral.com/1471-2407/10/179/prepub
